# c-MET/VEGFR-2 co-localisation impacts on survival following bevacizumab therapy in epithelial ovarian cancer: an exploratory biomarker study of the phase 3 ICON7 trial

**DOI:** 10.1186/s12916-022-02270-y

**Published:** 2022-02-11

**Authors:** Robert D. Morgan, Cristina Ferreras, Isabel Peset, Egle Avizienyte, Andrew G. Renehan, Richard J. Edmondson, Alexander D. Murphy, Shibani Nicum, Thomas Van Brussel, Andrew R. Clamp, Diether Lambrechts, Cong Zhou, Gordon C. Jayson

**Affiliations:** 1 Division of Cancer Sciences, Faculty of Biology, Health and Medicine, University of Manchester, Manchester, UK; 2 The Christie NHS Foundation Trust, Manchester, M20 4BX UK; 3 Medicines Discovery Catapult, Alderley Park, Cheshire, UK; 4 Cancer Communications and Consultancy Ltd., Knutsford, UK; 5 Manchester University NHS Foundation Trust, Manchester, UK; 6 Oxford University Hospitals NHS Foundation Trust, Oxford, UK; 7 Leuven Center for Cancer Biology, University of Leuven, Leuven, Belgium; 8 Cancer Biomarker Centre, Cancer Research UK Manchester Institute, Manchester, UK

**Keywords:** Epithelial ovarian cancer, Bevacizumab, c-MET, VEGFR-2, Single-nucleotide polymorphisms

## Abstract

**Introduction:**

Bevacizumab improves survival outcomes in women diagnosed with epithelial ovarian cancer (EOC). Pre-clinical data showed that the c-MET/VEGFR-2 heterocomplex negates VEGF inhibition through activation of c-MET signalling, leading to a more invasive and metastatic phenotype. We evaluated the clinical significance of c-MET and VEGFR-2 co-localisation and its association with VEGF pathway-related single nucleotide polymorphisms (SNPs) in women participating in the phase 3 trial, ICON7 (ClinicalTrials.gov identifier: NCT00262847).

**Materials and methods:**

Patients had FIGO stage I-IIA grade 3/poorly differentiated or clear cell carcinoma or stage IIB-IV epithelial ovarian, primary peritoneal or fallopian tube cancer. Immunofluorescence staining for co-localised c-MET and VEGFR-2 on tissue microarrays and genotyping of germline DNA from peripheral blood leukocytes for *VEGFA* and *VEGFR-2* SNPs was performed. The significance of these biomarkers was assessed against survival.

**Results:**

Tissue microarrays from 178 women underwent immunofluorescence staining. Multivariable analysis showed that greater c-MET/VEGFR-2 co-localisation predicted worse OS in patients treated with bevacizumab after adjusting for FIGO stage and debulking surgery outcome (hazard ratio [HR] 1.034, 95% confidence interval [95%CI] 1.010–1.059). Women in the c-MET/VEGFR-2^HIGH^ group treated with bevacizumab demonstrated significantly reduced OS (39.3 versus > 60 months; HR 2.00, 95%CI 1.08–3.72). Germline DNA from 449 women underwent genotyping. In the bevacizumab group, those women with the *VEGFR-2* rs2305945 G/G variant had a trend towards shorter PFS compared with G/T or T/T variants (18.3 versus 23.0 months; HR 0.74, 95%CI 0.53–1.03).

**Conclusions:**

In bevacizumab-treated women diagnosed with EOC, high c-MET/VEGFR-2 co-localisation on tumour tissue and the *VEGFR-2* rs2305945 G/G variant, which may be biologically related, were associated with worse survival outcomes.

**Supplementary Information:**

The online version contains supplementary material available at 10.1186/s12916-022-02270-y.

## Highlights

- Tissue microarrays and germline DNA from women recruited to the phase 3 trial, ICON7, underwent quantitative immunofluorescence for c-MET/VEGFR-2 co-expression and genetic sequencing for single nucleotide polymorphisms (SNPs) VEGF-pathway genes.
- High c-MET/VEGFR-2 co-localisation on tumour tissue independently predicted worse survival in bevacizumab-treated epithelial ovarian cancer.
- The *VEGFR-2* rs2305945 SNPs also independently predict worse survival in bevacizumab-treated epithelial ovarian cancer.

**Supplementary Information:**

The online version contains supplementary material available at 10.1186/s12916-022-02270-y.

## Introduction

Ovarian cancer is the commonest cause of gynaecological cancer-related death in Europe and North America [1]. Multi-modality first-line therapy includes cytoreductive surgery plus platinum-based chemotherapy, followed by maintenance therapies in certain subgroups [2]. One such maintenance therapy is the anti-angiogenic agent, bevacizumab, a humanised monoclonal antibody directed against vascular endothelial growth factor (VEGF) [3]. Two randomised phase 3 trials, ICON7 and GOG-0218, demonstrated significant improvements in progression-free survival (PFS) using bevacizumab as part of the first-line therapy, with improvements in overall survival (OS) in patients at the highest risk of relapse [4–7]. Although clinical and pathological features can be used to guide patient selection for first-line bevacizumab therapy, no molecularly defined predictive biomarkers have been validated. While we have identified plasma Tie2 as the first response biomarker for VEGF inhibitors, we were unable to identify a biomarker that would predict the benefit from bevacizumab [8–10].

In our initial study of pre-treatment biomarkers, a group of patients were identified who appeared to be disadvantaged by bevacizumab [8, 9]. The question is how such an effect can be induced by bevacizumab. A number of mechanisms have been described that account for tumour resistance to anti-angiogenic agents [11]. One involves the formation of a c-MET/VEGFR-2 heterocomplex [12]. VEGFR-2 and c-MET are receptor tyrosine kinases (RTKs) [13, 14]. VEGFR-2 dimerises the following binding to VEGF, and downstream RTK-mediated signalling leads to angiogenesis [13]. c-MET homodimerisation occurs following the binding of the hepatocyte growth factor (HGF) ligand, and RTK-mediated signalling leads to tumour proliferation and metastasis [14]. In a pre-clinical model of glioblastoma multiforme, Lu et al. demonstrated that following c-MET/VEGFR-2 heterocomplex formation, VEGF inhibits HGF/c-MET signalling by recruiting protein tyrosine phosphatase 1B (PTP1B) [12]. Thus, inhibition of VEGF using, for example, bevacizumab, negates the inhibitory effect of the c-MET/VEGFR-2 heterocomplex, thereby supporting HGF/c-MET signalling and driving tumorigenesis and progression [12]. To test the hypothesis that c-MET/VEGFR-2 heterocomplex formation is responsible for patients experiencing a worse outcome from bevacizumab, we tested the clinical significance of c-MET/VEGFR-2 co-localisation in epithelial ovarian cancer (EOC) and refined the analysis further by genotyping germline single nucleotide polymorphisms (SNPs) in VEGF pathway-related genes to assess whether specific SNPs were associated with c-MET/VEGFR-2 co-localisation and survival outcomes.

## Methodology

### Study participants

All participants provided written informed consent for the International Collaboration of Ovarian Neoplasms 7 (ICON7) trial (ClinicalTrials.gov number, NCT00262847): an international, randomised, multi-centre, open-label, phase 3 trial investigating the use of bevacizumab maintenance therapy as part of the first-line therapy in women diagnosed with high-risk FIGO (1988) stage I–IIA (grade 3/poorly differentiated tumours or clear cell carcinoma) or FIGO (1988) IIB-IV epithelial ovarian, primary peritoneal or fallopian tube cancer [5]. Progression-free survival (PFS) was defined as the time interval from the date of randomisation to the date of disease progression (defined clinically or radiologically, but not using CA125 criteria) or death. Overall survival (OS) was defined as the time interval from the date of randomisation to the date of death from any cause. All demographic and survival data were provided by the clinical trial centre co-ordinating ICON7 (Medical Research Council Clinical Trials Unit, University College London). As part of written informed consent, ICON7 participants could optionally agree to donate whole blood samples and pre-chemotherapy tumour tissue for translational research. Data presented here is from all patients who agreed to donate tumour tissue and/or whole blood samples. No case selection has been carried out.

### Immunofluorescence biomarkers

Tissue microarrays (TMAs) from EOC tumour tissue sections underwent immunofluorescent staining using the BenchMark ULTRA system according to the RUO DISCOVERY universal staining procedure (Ventana Medical Systems). TMA sections were deparaffinised at 69°C for 8 min per cycle. Antigen retrieval occurred by pre-treatment with Cell Conditioning Solution 2 (CC2) (Ventana Medical Systems) and incubation at 95 °C for 4, 8, 16, 24 and 32 min. TMA sections were then blocked by incubating with DISCOVERY inhibitor (Ventana Medical Systems) for 8 min. Sections were warmed to 37 °C and then incubated with the following primary antibodies: VEGF receptor 2 (55B11) rabbit monoclonal antibody (dilution 1:600; Cell Signalling Technology) and c-MET (1G7NB) mouse monoclonal antibody (dilution 1:300; Novus Biologicals) for 60 min. Sections were then incubated with the corresponding HRP-conjugated secondary antibodies for 8 min. The Tyramide Signal Amplification (PerkinElmer) was used on all slides to improve visualisation following incubation with the secondary antibody. Tyramide labelled with Cy3 (to visualise VEGFR-2, dilution 1:50; PerkinElmer) or FITC (to visualise c-MET, dilution 1:50; PerkinElmer) diluted in 1X Plus Amplification Diluent (PerkinElmer) were used.

Following the first staining, the second staining on the same section was performed using anti-human collagen type IV (dilution 1:300; Merck Millipore) to identify the stroma in each core. Sections were pre-treated with CC2 at 95 °C for 4 min and then 8 min. Slides were then cooled to 37 °C before the second primary antibody was applied manually, followed by incubation for 60 min. Slides were then incubated with a corresponding HRP-conjugated secondary antibody for 16 min followed by incubation with the Cy5 fluorophore-conjugated tyramide (dilution 1:50) diluted in 1X Plus Amplification Diluent for 8 min. Slides were removed from the instrument and washed twice in EZ Prep buffer (Ventana Medical Systems). SlowFade Gold Antifade Mountant with 4′,6-diamidino-2-phenylindole (DAPI) was added, and slides were mounted with coverslips.

Leica Aperio VERSA-200 digital pathology scanner was used to visualise immunofluorescence staining. Images were acquired at × 20 magnification using the Andor iXon 888 camera (Additional File 1: Fig. S1). Immunofluorescence images from TMA sections were analysed by molecular pathology image analysis using Definiens Tissue Studio 2.7. Each TMA core was segmented into tumour and stroma by machine learning using collagen IV staining to identify stroma. VEGFR-2 staining was used to detect vessels, and to calculate number of vessels, vessels density (number of vessels per tumour area), median vessel size and VEGFR-2 expression (mean of stain intensity) in the tumour component of each TMA core. c-MET/VEGFR-2 co-localisation was quantified using c-MET total intensity in VEGFR-2 positive vessel. The co-localisation values were not standardised by tumour area, vessel number and sizes because this information was included separately in data analysis.

### Genotyping biomarkers

Peripheral blood samples were collected in K2EDTA blood tubes. Germline DNA was extracted from the precipitated leukocyte cell fraction. Genotyping was performed at the VIB Center for Cancer Biology (Leuven, Belgium) using the Sequenom MassARRAY® system (iPLEX GOLD) as reported previously [15]. Single nucleotide polymorphisms (SNPs) in *VEGF-A* and *VEGFR-2* were analysed. A detailed description of the way in which SNPs were selected in these genes has been published previously [15, 16].

### Statistical power consideration

In the ICON7 trial, bevacizumab maintenance therapy showed an PFS advantage of hazard ratio (HR) 0.81 compared to placebo [5]. A group of patients were however identified whom appeared to be disadvantaged by bevacizumab [8, 9]. The HR between those patients whom benefitted versus those that did not was assumed to be 0.56, a 30% reduction from the original HR of 0.81. Based on a two-sided Cox proportional hazard regression analysis, it was estimated that a total number of 124 patients will be needed to achieve a significance level of 0.05 and a power of 0.8, assuming data censorship was less than 25%. Including a 15% contingency cohort, a minimum patient size of 143 would therefore be required. In total, samples from all 178 patients who gave consent to donate tumour tissue were employed in this study.

### Statistical analysis

Variables from three types of data were analysed in this study: (1) clinical data, (2) immunofluorescence biomarker data including VEGFR-2 and c-MET expression and (3) genotyping biomarker data for SNPs in *VEGF-A* and *VEGFR-2*. The analysis started from investigating the associations between immunofluorescence variables and treatment outcomes (data types 1 and 2), followed by identifying SNPs that were associated with selected immunofluorescence biomarkers in matched patients (data types 2 and 3) and finally exploring the association between the identified SNPs and treatment outcomes (data types 1 and 3).

The relationship between variables in clinical, immunofluorescence and genotyping data were assessed using one-way ANOVA for continuous variables or chi-squared tests for categorical variables.

The pre-treatment prognostic and predictive significance of each variable were assessed by including the biomarker as a sole covariate (univariable) in a proportional hazards model for PFS or OS. Here, prognostic significance refers to whether there is a significant association between a biomarker and survival independent of treatment arms, while predictive significance refers to if the association between a biomarker and survival are significantly different depending on treatment arms. Variables were included in continuous form where possible and were subject to transformation, such as log_2_ or dichotomisation by median. Assumption of proportionality was verified based on Schoenfeld residuals [17]. Martingale residuals from each marker specific analysis was examined for evidence of nonlinearity in the biomarker-hazard relationship [18]. Candidate biomarkers with *P*-values ≤ 0.05 in the univariable analysis were selected for subsequent multivariable proportional hazard regression analysis. A backward stepwise method was applied to identify the optimum subset of variables that associated with PFS or OS. Interactions between prognostic biomarkers of interest and treatment arms were included to seek predictive biomarkers that predicted benefit from bevacizumab. To address potential confounding factors, clinical variables that are significantly associated with immunofluorescence biomarkers are included in multivariable models regardless of their association with patient survival. Their interaction with immunofluorescence biomarkers were also interrogated.

Genotyping data from *VEGF-A* and *VEGFR-2* were introduced to explore potential biological mechanisms underpinning the immunofluorescence data. The SNPs were filtered by their association with immunofluorescence biomarkers and those that strongly associated were investigated for their prognostic and predictive significance with respect to PFS/OS using the proportional hazard model described above. Analyses were carried out in accordance with the REMARK guidelines [19] and were implemented using R 3.6.0 [20].

## Results

### Immunofluorescence biomarkers

To test the clinical significance of c-MET/VEGFR-2 co-localisation in samples from 178 patients diagnosed with EOC, a TMA-based immunofluorescence staining method was developed for c-MET and VEGFR-2. The statistics of key immunofluorescence biomarkers are summarised in Additional File 2: Table S1. The clinical data from these 178 cases was similar to the original ICON7 population with a slightly higher proportion of clear cell carcinoma cases in the immunofluorescence biomarker study, although the difference was not significant (Fisher’s exact test, *P* = 0.07) (Table 1). In this group of 178 patients, those with FIGO stage III/IV disease demonstrated significantly higher vessel density (*P* = 0.005), number of vessels (*P* = 0.004), median vessel size (*P* = 0.006) and VEGFR-2 expression (*P* = 0.003); consistent with the concept that angiogenesis is associated with tumour growth and metastasis (Additional File 2: Table S1).

**Table 1:** Demographic data. Data is presented as median (range) or number (percentage). *C* carboplatin, *P* paclitaxel, *B* bevacizumab

Clinical data	Immunofluorescence biomarker study (***n*** = 178)		Genotyping biomarker study (***N*** = 449)		ICON7 population (***N*** = 1528)	
	C+P (***N*** = 91)	C+P+B (***N*** = 87)	C+P (***N*** = 216)	C+P+B (***N*** = 233)	C+P (***N*** = 764)	C+P+B (***N*** = 764)
**Age, years**	58 (35–76)	56 (25–75)	58 (24–79)	56 (24–77)	57 (18–81)	57 (24–82)
**ECOG performance status**						
0	32 (35)	38 (44)	87 (40)	105 (45)	358 (47)	334 (44)
1	52 (57)	45 (52)	111 (51)	117 (50)	354 (47)	366 (48)
2	2 (2)	2 (2)	13 (6)	7 (3)	43 (6)	45 (6)
Unknown	5 (5)	2 (2)	5 (2)	4 (2)	9 (1)	19 (2)
**Histological subtype**						
Serous	61 (67)	56 (64)	148 (69)	158 (68)	529 (69)	525 (69)
Mucinous	0	1 (1)	3 (1)	2 (1)	15 (2)	19 (2)
Endometrioid	8 (9)	4 (5)	17 (8)	15 (6)	57 (7)	60 (8)
Clear cell	14 (15)	15 (16)	23 (11)	30 (13)	60 (8)	67 (9)
Mixed	5 (5)	10 (11)	13 (6)	21 (9)	48 (6)	50 (5)
Others	3 (3)	1 (1)	12 (6)	7 (3)	55 (7)	53 (7)
**Histological grade**						
1 (well differentiated)	2 (2)	2 (2)	10 (5)	12 (5)	57 (7)	41 (5)
2 (moderately differentiated)	13 (14)	11 (13)	34 (16)	39 (17)	142 (19)	175 (23)
3 (poorly differentiated)	76 (84)	73 (84)	169 (78)	179 (77)	556 (74)	538 (71)
Unknown	0	1 (1)	3 (1)	3 (1)	10	10
**FIGO (1988) stage**						
I/II	17 (19)	22 (25)	46 (21)	44 (19)	145 (19)	137 (18)
III/IV	74 (81)	65 (75)	170 (79)	189 (81)	619 (81)	627 (82)
**Debulking surgery**						
> 1 cm residual disease	35 (38)	26 (30)	62 (29)	61 (26)	195 (26)	192 (26)
≤ 1 cm residual disease	56 (62)	61 (70)	143 (71)	167 (74)	569 (76)	572 (76)

The association between vascular tissue markers of angiogenesis and higher FIGO stage provided confidence in the validity of the immunofluorescence biomarkers and sample set, supporting further evaluation of the relationship between immunofluorescence biomarkers and survival outcomes. As a first step, a univariable analysis of clinical prognostic factors and vascular tissue biomarkers was performed (Additional File 2: Table S2). Both FIGO stage (III/IV vs. I/II) and amount of residual disease (≤ 1 cm vs. > 1 cm) following debulking surgery were of prognostic significance, in keeping with other ovarian cancer datasets (Additional File 2: Table S2) [6, 7]. In addition, number of vessels (*P* = 0.015) and VEGFR-2 expression (*P* = 0.027) inversely associated with PFS regardless of treatment (Additional File 2: Table S2). Importantly, in this univariable analysis, c-MET/VEGFR-2 co-localisation predicted significantly worse OS (*P* = 0.001) in patients treated with bevacizumab (Additional File 2: Table S2).

**Fig. 1:**
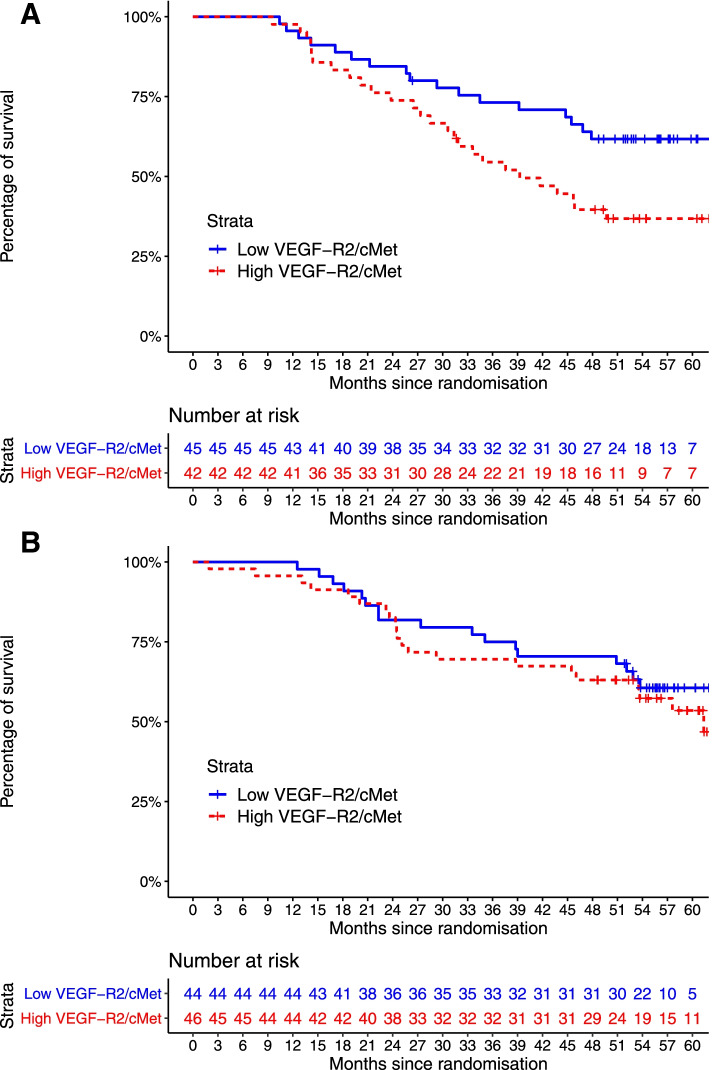
**A** Kaplan-Meier estimates of overall survival in patients (*n* = 87) treated with carboplatin/paclitaxel plus bevacizumab (experimental group). Patients are dichotomised by their median average expression of c-MET/VEGFR-2 into c-MET/VEGFR-2^HIGH^ (*n* = 42) and c-MET/VEGFR-2^LOW^ (*n* = 45) groups. The median OS was 39.3 months for c-MET/VEGFR-2^HIGH^ and >60 months c-MET/VEGFR-2^LOW^ (HR 2.00, *P* = 0.03). **B** Kaplan-Meier estimates of overall survival in patients (n=90) treated with carboplatin/paclitaxel (control group). Patients are dichotomised by their median average expression of c-MET/VEGFR-2 into c-MET/VEGFR-2^HIGH^ (*n* = 46) and c-MET/VEGFR-2^LOW^ (*n* = 44) groups. Both groups had a median OS > 60 months.

We next carried out a multivariable analysis to interrogate whether immunofluorescence biomarkers could predict treatment benefit of bevacizumab, but not placebo, with established prognostic clinical factors. Consistent with other ovarian cancer studies, FIGO stage (III/IV vs. I/II; hazard ratio [HR] 2.384, 95% confidence interval [95%CI] 1.181–4.815, *P* = 0.015) and amount of residual disease (≤ 1 cm vs. > 1 cm; HR 0.243, 95%CI 0.147–0.399, *P* < 0.001) were significant prognostic factors for PFS (Table 2). For OS, the amount of residual disease was also significant (≤ 1 cm vs. > 1 cm; HR 0.397, 95%CI 0.228–0.694, *P* = 0.001; Table 2). Multivariable analysis showed that greater c-MET/VEGFR-2 co-localisation predicted worse OS in patients treated with bevacizumab after adjusting for FIGO stage and debulking surgery outcome (HR 1.034, 95%CI 1.010–1.059, *P* = 0.006) (Table 2). Furthermore, by dichotomising patients according to the median expression of c-MET/VEGFR-2 into c-MET/VEGFR-2^LOW^ (≤ median) and c-MET/VEGFR-2^HIGH^ (> median) groups, data showed that patients with c-MET/VEGFR-2^HIGH^ tumours treated with bevacizumab had significantly reduced OS compared to c-MET/VEGFR-2^LOW^ tumours (39.3 vs. > 60 months; multivariable HR 2.00, 95%CI 1.08–3.72, *P* = 0.03; Fig. 1A) whereas in the placebo group, there was no significant difference (multivariable HR 1.26, 95%CI 0.67–2.39, *P* = 0.47; Fig. 1B).

**Table 2:** Multivariable survival analysis for immunofluorescence biomarkers. *95%CI* 95% confidence interval, *HR* hazard ratio, *PFS* progression-free survival, *OS* overall survival. In the multivariable analysis, a *P*-value cut-off of ≤ 0.05 was considered statistically significant. Predictive model, a model exploring if the association between a biomarker and survival are significantly different depending on treatment arms: HR, 95%CI and *P*-values come from the interaction term. Clinical factors prognostic for PFS/OS were included in the model

Covariate name	Predictive model					
	PFS			OS		
	HR	95%CI	***P***-value	HR	95%CI	***P***-value
***Clinical factors***						
FIGO stage (III/IV vs. I/II)	2.384	1.181–4.815	**0.015**	1.994	0.893–4.454	0.092
Debulking surgery outcome (≤ 1 cm vs. > 1 cm residual disease)	0.243	0.147–0.399	**< 0.001**	0.397	0.227–0.695	**0.001**
***Immunofluorescence biomarker***						
c-MET/VEGFR-2 co-localisation (increase of expression)	1.011	1.004–1.018	**0.003**	1.001	0.995–1.008	0.671
Bevacizumab arm	–	–	ns	1.116	0.995–1.008	0.669
***Predictive significance***						
c-MET/VEGFR-2 co-localisation in bevacizumab arm (interaction)	–	–	ns	1.034	1.010–1.059	**0.006**

### Genotyping biomarker

**Fig. 2:**
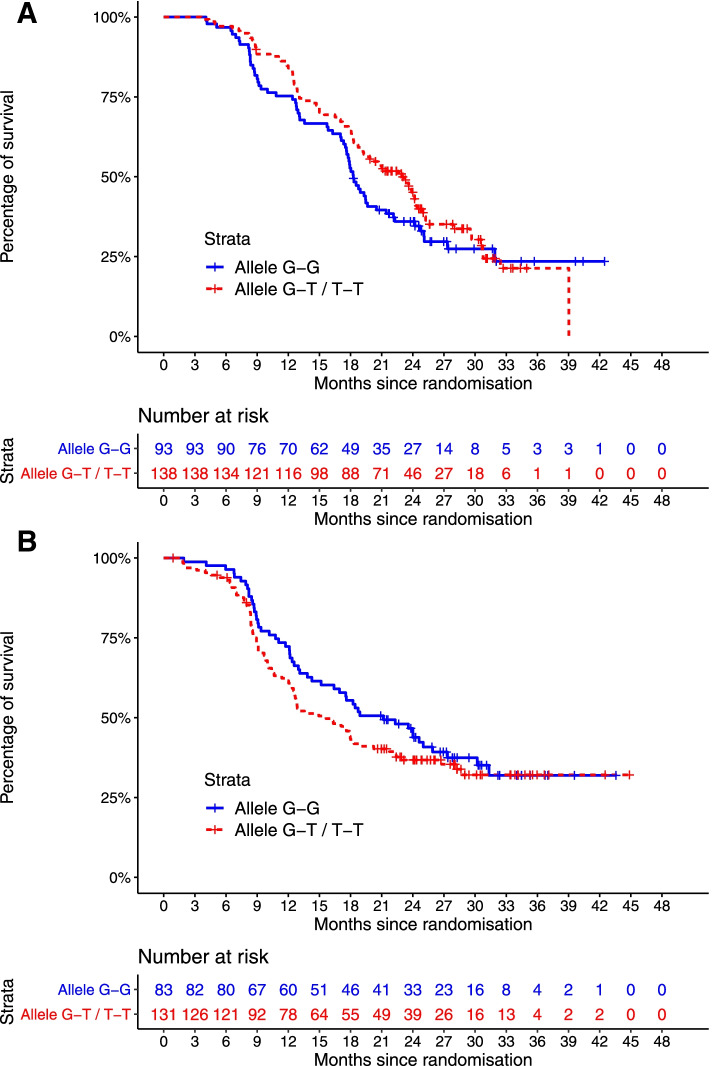
**A** Kaplan-Meier estimates of progression-free survival in patients (*n* = 231) treated with carboplatin/paclitaxel plus bevacizumab (experimental group). Patients are separated into two groups: those with the *VEGFR-2* rs2305945 G/G variant (*n* = 93) and those with the *VEGFR-2* rs2305945 G/T or T/T variant (*n* = 138). The median PFS was 18.2 months for those patients with the *VEGFR-2* rs2305945 G/G variant and 23.0 months for those with the *VEGFR-2* rs2305945 G/T or T/T variant (HR 0.74, *P* = 0.07). **B** Kaplan-Meier estimates of progression-free survival in patients (*n* = 214) treated with carboplatin/paclitaxel (control group). Patients are separated into two groups: those with the *VEGFR-2* rs2305945 G/G variant (*n* = 83) and those with the *VEGFR-2* rs2305945 G/T or T/T variant (*n* = 131). The median PFS was 21.2 months for those patients with the *VEGFR-2* rs2305945 G/G variant and 15.6 months for those with the *VEGFR-2* rs2305945 G/T or T/T variant (HR 1.14, *P* = 0.48)

Four hundred and forty-nine patients underwent SNP genotyping: 216 in the control group and 233 in the bevacizumab group (Table 1). Thirty-five *VEGF-A* and *VEGFR-2* SNPs were investigated to understand the potential genetic mechanisms underpinning the variation in c-MET/VEGFR-2 co-localisation (Additional File 2: Table S3). Focusing on the 142 matched patients who underwent both immunofluorescence studies and genotyping, three SNPs were identified that were strongly associated with high co-localisation of c-MET and VEGFR-2. These included *VEGF-A* rs3025033 (G variant; chi-squared test, *P* = 0.138), *VEGFR-2* rs1870377 (A variant; chi-squared test, *P* = 0.151) and *VEGFR*-2 rs2305945 (G variant; chi-squared test, *P* = 0.047) (Additional File 2: Table S3). The prognostic and predictive significance of these SNPs were evaluated, with univariable and multivariable results presented in Additional File 2: Table S3 and Table 3, respectively. Of these three SNPs, *VEGFR-2* rs2305945 independently predicted PFS in the bevacizumab-treated cohort after adjusting for known prognostic clinical factors (multivariable HR 1.592, 95%CI 1.110–2.284, *P* = 0.012) (Table 3). Patients with the *VEGFR-2* rs2305945 G/G variant who were treated with bevacizumab had shorter PFS than those with G/T or T/T variants (18.3 vs. 23.0 months; HR 0.74, 95%CI 0.53–1.03, *P* = 0.07; Fig. 2A). In contrast, there was moderately longer PFS in patients with the *VEGFR-2* rs2305945 G/G variant who were treated with chemotherapy alone (21.2 [G/G variant] vs. 15.6 months [G/T or T/T variants]; HR 1.14, 95% 0.79–1.63, *P* = 0.48; Fig. 2B). The frequency of each variant in the three key SNPs are shown in Additional File 2: Table S4. The Kaplan-Meier curves of PFS for *VEGF-A* rs3025033 and *VEGFR-2* rs1870377 SNPs are shown in Additional File 2: Fig. S2.

**Table 3:** Multivariable survival analysis for genotype biomarkers. *G allele. *95%CI* 95% confidence interval, *HR* hazard ratio, *RD* residual disease, *PFS* progression-free survival, *OS* overall survival. In the multivariable analysis, a *P*-value cut-off of ≤ 0.05 was considered statistically significant. Predictive model, a model exploring if the association between a biomarker and survival are significantly different depending on treatment arms: HR, 95%CI and *P*-values come from the interaction term. Clinical factors prognostic for PFS/OS were included in the model

Covariate name	Predictive model					
	PFS			OS		
	HR	95%CI	***P***-value	HR	95%CI	***P***-value
***Clinical biomarker***						
FIGO stage (III/IV vs. I/II)	3.171	2.118–4.747	< 0.001	2.434	1.409–4.205	0.001
Debulking surgery outcome (≤ 1 cm vs. > 1 cm RD)	0.506	0.394–0.651	< 0.001	0.396	0.274–0.573	< 0.001
***Genotype biomarker***						
rs2305945 (*VEGFR-2*)*	0.794	0.961–1.650	0.095	–	–	ns
Bevacizumab	0.497	0.296–0.833	0.008	–	–	ns
***Predictive significance***						
rs2305945 (*VEGFR-2*) in the bevacizumab arm* (interaction)	1.592	1.110–2.284	**0.012**	–	–	ns

### Immunofluorescence and genotyping biomarkers

The two multivariable analyses show that c-MET/VEGFR-2 co-localisation (178 patients) and *VEGFR-2* rs2305945 (449 patients) are independent predictive factors for bevacizumab-associated outcomes, supporting the hypothesis that there are a group of patients whose outcome is adversely affected by treating with bevacizumab. Furthermore, the *VEGFR-2* rs2305945 G/G variant is associated with increasing c-MET/VEGFR-2 co-localisation (*P* = 0.047; Additional File 2: Table S3) and each biomarker predicts worse PFS (*VEGFR-2* rs2305945: HR 1.592, *P* = 0.012; Table 3) and OS (c-MET/VEGFR-2 co-localisation: HR 1.034, *P* = 0.006; Table 2) in patients treated with bevacizumab, suggesting a possible biological and/or clinical interaction (Additional File 1: Fig. S3). An exploratory analysis on the relationship between c-MET/VEGFR-2 co-localisation and the *VEGFR-2* rs2305945 SNP was carried out by investigating their interaction in one multivariable model (Additional File 2: Table S5). It was observed that patients had worse PFS and OS if they had high c-MET/VEGFR-2 co-localisation and the *VEGFR-2* rs2305945 G/G variant, although the difference was not significant (*P* = 0.071 for PFS and *P* = 0.272 for OS; Additional File 2: Table S5).

## Discussion

In our initial studies of biomarkers that predict benefit from bevacizumab in ovarian cancer, we identified a group of patients who appeared to be disadvantaged by bevacizumab [8, 9]. The question was what potential mechanism(s) could have been responsible for this observation? A contemporaneous publication had identified a mechanism in which c-MET and VEGFR-2 were fused in a heterocomplex and subsequent VEGF inhibitor-mediated inhibition of VEGFR-2 activated c-MET signalling by inhibition of the phosphatase, PTP1B [12]. Thus, we hypothesised that expression of the c-MET/VEGFR-2 heterocomplex might account for the adverse outcome of a subgroup of bevacizumab-treated patients. Here, by using immunofluorescence image analysis studies to detect co-localisation of c-MET and VEGFR-2 as a surrogate for heterocomplex expression, we have shown that co-localisation, particularly in the context of the *VEGFR-2* rs2305945 G/G variant, predicted a worse outcome following bevacizumab treatment. These two multivariable analyses show that c-MET/VEGFR-2 co-localisation and the *VEGFR-2* rs2305945 G/G variant are independent predictive factors for bevacizumab-associated outcomes; supporting the hypothesis that there are a group of patients whose outcome is adversely affected through treatment with bevacizumab.

The immunofluorescence image analysis reported here provides the first clinical data from a prospective clinical trial supporting c-MET/VEGFR-2 heterocomplex formation as a putative resistance mechanism against VEGF inhibition [12]. The interesting finding that higher levels of c-MET/VEGFR-2 co-localisation were associated with worse OS provides evidence to support the use of alternative therapies for such patients; in particular, targeted agents that inhibit both c-MET and VEGFR-2 kinase domains.

The HGF/c-MET axis can be inhibited through small molecule tyrosine-kinase inhibitors or monoclonal antibodies directed against either c-MET or HGF. Two oral small-molecule multi-kinase inhibitors with activity against c-MET: cabozantinib [21] and crizotinib [22], are effective in renal, thyroid and lung cancer [23–25]. Cabozantinib is active against both c-MET and VEGFR-2 and could be considered in future trials recruiting patients diagnosed with EOC where their tumour manifests co-localisation of c-MET/VEGFR-2. Indeed, three phase II trials with cabozantinib have reported modest activity in chemo-resistant ovarian cancer, although none of the trials incorporated a biomarker [26–28].

We were interested in VEGF pathway-related SNPs that pertained to the c-MET/VEGFR-2 heterocomplex. The *VEGFR-2* rs2305945 G/G variant was found to be significantly associated with c-MET/VEGFR-2 co-localisation and while the co-localisation predicted worse outcome from bevacizumab in OS, the SNP predicted worse outcome from bevacizumab in PFS. Worse outcomes from bevacizumab, though not significant, were observed in patients whose tumours manifested both c-MET/VEGFR-2 co-localisation and *VEGFR-2* rs2305945 G/G variant. However, it should be noted that this analysis was carried out in a small cohort of bevacizumab-treated patients with both immunofluorescence and genotyping data available and evaluation of the impact of the two factors on survival is confounded by the potential that the SNP could influence c-MET/VEGFR-2 heterocomplex formation.

Accepting that there is an association between *VEGFR-2* rs2305945 and c-MET/VEGFR-2 expression, the question is whether there is any evidence for a biological role of this SNP in the c-MET/VEGFR-2 heterocomplex? Although this SNP is located within a non-coding region of *VEGFR-2*, there are data suggesting it is of clinical interest. Indeed, previous data has reported that rs2305945 is associated with differential response to VEGF inhibitors in age-related macular degeneration and also protective against ovarian hyperstimulation syndrome [29, 30]. The latter is of particular relevance here given the physiological importance of VEGF to follicular cysts formation and the plethora of trials that have demonstrated the clinical activity of VEGF inhibitors in ovarian cancer, irrespective of the platinum/progression-free interval.

The main strength of the clinical data reported in this study is that it was prospectively collected, although it represents only a proportion of the patients recruited to ICON7. In addition, genotyping biomarker assessment was performed using an established methodology [15, 16]. As an exploratory study for biomarker discovery, no correction was made for multiple testing in the analysis; instead, chance of false discovery was controlled by verifying against biological rationale. For example, only SNPs from the VEGF pathway were chosen for analysis because of their putative molecular relevance to bevacizumab and the VEGF-A/VEGFR-2 signalling pathway. These SNPs were filtered by their association with the immunofluorescence biomarkers before carrying out survival analysis. Further clinical studies will be needed to corroborate the findings, but confidence in the results can be gained from the similarity in behaviour of conventional prognostic factors with the main ICON7 study [5]. Moreover, this study and the translational work from GOG-0218 reported the potential predictive value of vessel density for bevacizumab-treated patients [31].

The main limitation of our study is the sample size, with 11.6% (178/1528) and 29.4% (449/1528) of women from ICON7 donating tumour tissue and/or blood, respectively. It is notable however, that donation of research tumour tissue and/or blood samples was optional, and not mandated within the trial protocol. Therefore, the sample size reflects the proportion of women willing to donate translational research samples. Moreover, the number required to detect differences in c-MET/VEGFR-2 co-localisation, according to the statistical power consideration, was achieved in the study. Another limitation of this biomarker study is the absence of an independent validation cohort and this could be assessed in an appropriately powered prospective study.

In conclusion, in bevacizumab-treated patients diagnosed with EOC, high c-MET/VEGFR-2 co-localisation on tumour tissue and the *VEGFR-2* rs2305945 G/G variant, which may be biologically related, was associated with worse survival outcomes. In patients who did not receive bevacizumab, high c-MET/VEGFR-2 co-localisation showed no association with outcome.

## Supplementary Information

**Additional file 1: Figure S1**- Examples of tissue microarray immunofluorescence staining for VEGFR-2 and c-MET. **Figure S2**- Progression-free survival for *VEGF-A* rs3025033 and *VEGFR-2* rs1870377 SNPs in the control (chemotherapy) and experimental (chemotherapy plus bevacizumab) group. **Figure S3**- Summary of findings for c-MET/VEGFR-2 co-localisation and SNP *VEGFR-2* rs2305945.

**Additional file 2: Table S1**- Characteristics of immunofluorescence biomarkers and their association with demographic data. **Table S2**- Prognostic and predictive biomarkers assessed as significant in the univariable analysis. **Table S3**- List of the 35 SNPs in *VEGF-A* and *VEGFR-2* tested and their association with c-MET/VEGFR-2 co-localisation. **Table S4**- Frequency of three key SNPs in VEGF-related genes. **Table S5**- Relationship between c-MET/VEGFR-2 co-localisation and *VEGFR-2* rs2305945 SNP.

## Data Availability

All information regarding patients is strictly confidential.
