# Supplementary material for: c-MET/VEGFR-2 co-localisation impacts on survival following bevacizumab therapy in epithelial ovarian cancer: an exploratory biomarker study of the phase 3 ICON7 trial
Source: BMC Med. 2022 Feb 11;20:59. doi: 10.1186/s12916-022-02270-y (PMC8832801; doi:10.1186/s12916-022-02270-y)
Supplement: Supplementary file 1 — Additional file 1: Figure S1- Examples of tissue microarray immunofluorescence staining for VEGFR-2 and c-MET. Figure S2- Progression-free survival for VEGF-A rs3025033 and VEGFR-2 rs1870377 SNPs in the control (chemotherapy) and experimental (chemotherapy plus bevacizumab) group. Figure S3- Summary of findings for c-MET/VEGFR-2 co-localisation and SNP VEGFR-2 rs2305945. [file 12916_2022_2270_MOESM1_ESM.pdf]

Supplementary Figure 1: Examples of tissue microarray immunofluorescence staining for VEGFR-2 and c-MET.

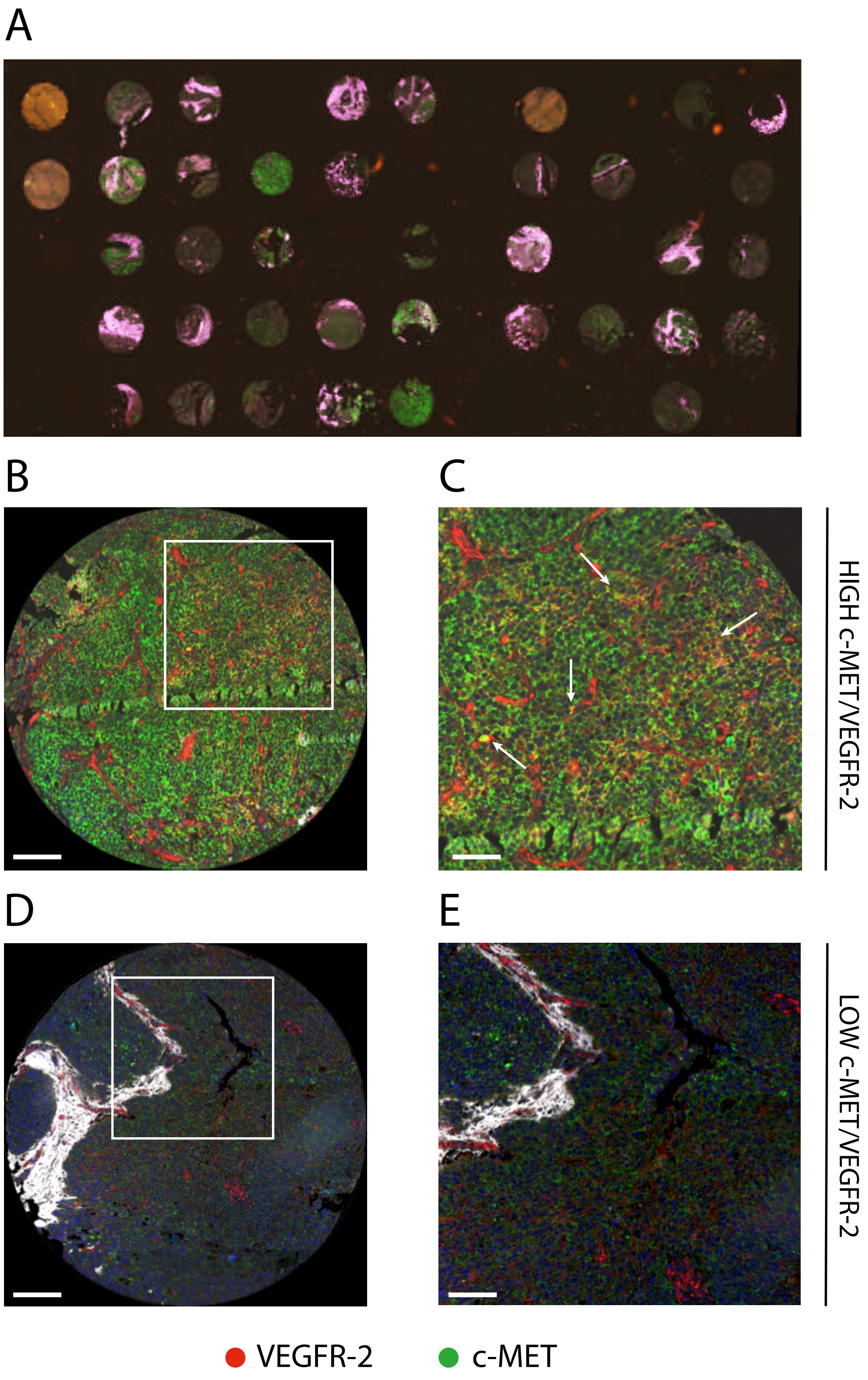

Key: VEGFR-2, red staining; c-MET, green staining; VEGFR-2/c-MET co-localisation, orange staining and white arrows; white is stroma stained by collagen IV and blue nuclei by DAPI. Supplementary Figure 1B and 1D- white line, 200μM; Supplementary Figure 1B and 1D- white box (insert) refers to magnified image shown in Figure 1C and 1E, respectively; Supplementary Figure 1C & 1E- white line, 100μM.

Supplementary Figure 2: Progression-free survival for SNPs VEGF-A rs3025033 (A-B) and VEGFR-2 rs1870377 (C-D) in the chemotherapy group (A/C) and chemotherapy plus bevacizumab group (B/D).

A

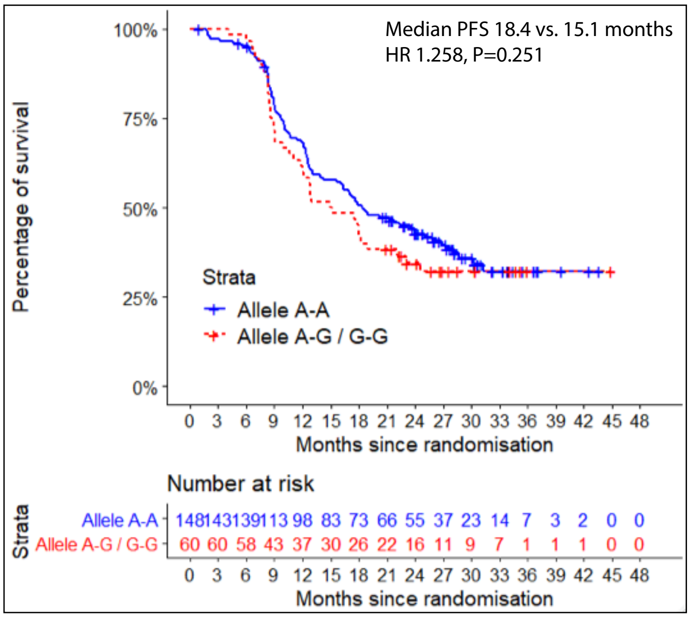

VEGF-A rs3025033 - chemotherapy (control group)

B

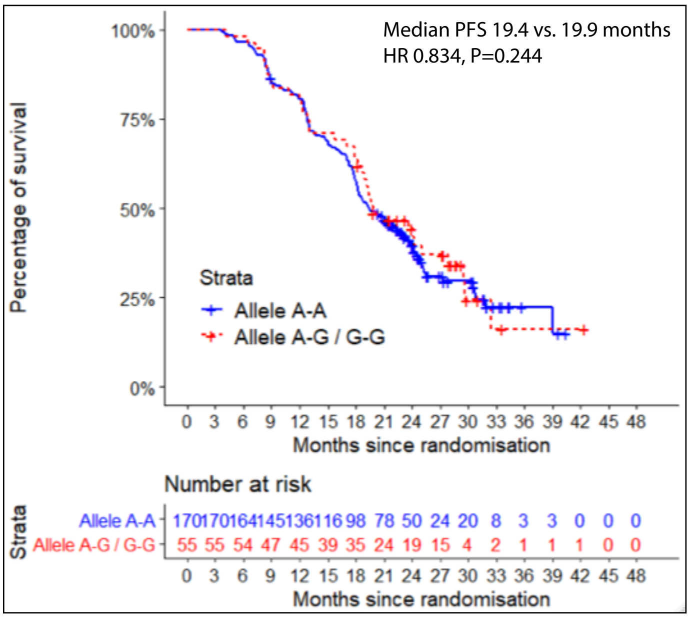

VEGF-A rs3025033 - chemotherapy + bevacizumab (experimental group)

C

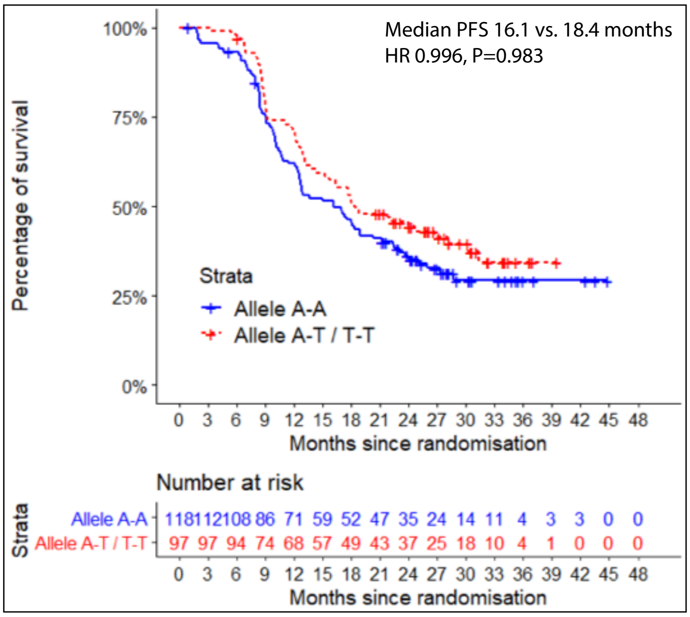

VEGFR-2 rs1870377 - chemotherapy (control group)

D

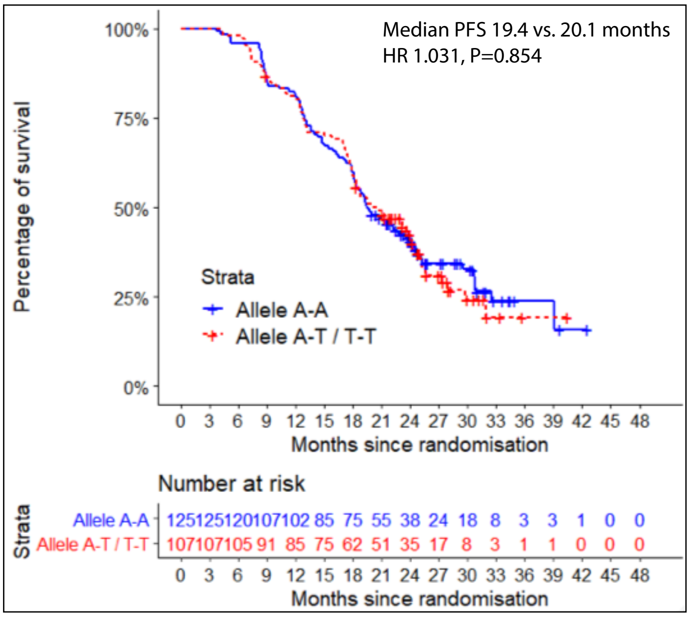

VEGFR-2 rs1870377 - chemotherapy + bevacizumab (experimental group)

Supplementary Figure 3: Summary of findings for c-MET/VEGFR-2 co-localisation and SNP VEGFR-2 rs2305945.

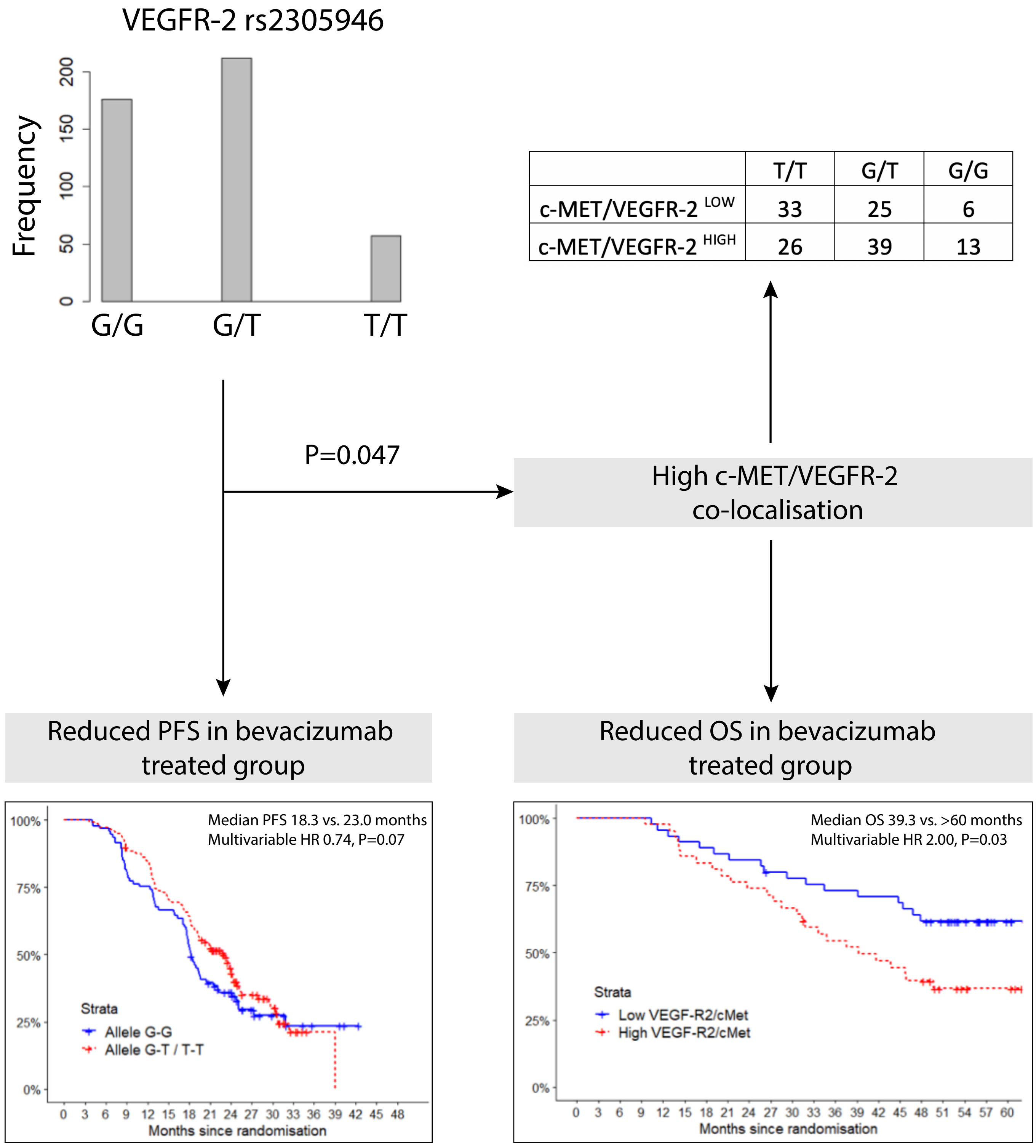

VEGFR-2 rs2305945 - chemotherapy + bevacizumab (experimental group)

c-MET/VEGFR-2 co-localisation - chemotherapy + bevacizumab (experimental group)
