# Supplementary material for: c-MET/VEGFR-2 co-localisation impacts on survival following bevacizumab therapy in epithelial ovarian cancer: an exploratory biomarker study of the phase 3 ICON7 trial
Source: BMC Med. 2022 Feb 11;20:59. doi: 10.1186/s12916-022-02270-y (PMC8832801; doi:10.1186/s12916-022-02270-y)
Supplement: Supplementary file 2 — Additional file 2: Table S1- Characteristics of immunofluorescence biomarkers and their association with demographic data. Table S2- Prognostic and predictive biomarkers assessed as significant in the univariable analysis. Table S3- List of the 35 SNPs in VEGF-A and VEGFR-2 tested and their association with c-MET/VEGFR-2 co-localisation. Table S4- Frequency of three key SNPs in VEGF-related genes. Table S5- Relationship between c-MET/VEGFR-2 co-localisation and VEGFR-2 rs2305945 SNP. [file 12916_2022_2270_MOESM2_ESM.docx]

| **Imaging features** | **Experimental arm** | | | | | **Standard arm** | | | |
| --- | --- | --- | --- | --- | --- | --- | --- | --- | --- |
|  | **Median** | | **IQR** | | | **Median** | | | **IQR** |
| Vessel density | 8.71 | | 22.78 | | | 8.31 | | | 27.11 |
| Number of vessels | 14 | | 27 | | | 12 | | | 34 |
| Median vessel size | 89.00 | | 49.24 | | | 91.50 | | | 57.47 |
| VEGFR-2 expression | 628.24 | | 1277.49 | | | 531.36 | | | 1804.88 |
| c-MET/VEGFR-2 co-localisation | 212.72 | | 772.56 | | | 255.83 | | | 1091.95 |
|  | | | | | | | | | |
| **Imaging features** | **Association with demographic data (p-values)** | | | | | | | | |
|  | **Age** | **ECOG PS** | | **Histology** | **Grade** | | **FIGO stage**  **(III/IV vs. I/II)** | **Debulking surgery**  **(≤1cm vs. >1cm RD)** | |
| Vessel density | 0.716 | 0.644 | | 0.423 | 0.228 | | 0.005 | 0.163 | |
| Number of vessels | 0.672 | 0.525 | | 0.256 | 0.529 | | 0.004 | 0.093 | |
| Median vessel size | 0.424 | 0.583 | | 0.069 | 0.329 | | 0.006 | 0.129 | |
| VEGFR-2 expression | 0.824 | 0.542 | | 0.174 | 0.694 | | 0.003 | 0.065 | |
| c-MET/VEGFR-2 co-localisation | 0.892 | 0.603 | | 0.305 | 0.698 | | 0.014 | 0.119 | |

**Supplementary Table 1. Characteristics of immunofluorescence biomarkers and their association with demographic data.** The descriptive statistics of the immunofluorescence biomarkers are summarised in the upper part of the table and the p-values of their association with demographic data are summarised in the bottom table. Key: ECOG PS, Eastern Cooperative Oncology Group performance status; FIGO, The International Federation of Gynaecology and Obstetrics; IQR, inter-quartile range; RD, residual disease. A p-value cut off of ≤0.05 was applied to determine statistical significance.

| **Covariate name** | **Prognostic model** | | | | **Predictive model** | | | |
| --- | --- | --- | --- | --- | --- | --- | --- | --- |
|  | **PFS** | | **OS** | | **PFS** | | **OS** | |
|  | HR | P-value | HR | P-value | HR | P-value | HR | P-value |
| ***Clinical data*** |  |  |  |  |  |  |  |  |
| Age* | 1.165 | 0.666 | 1.730 | 0.136 | 1.623 | 0.504 | 1.306 | 0.769 |
| ECOG performance status | 1.243 | 0.273 | 1.124 | 0.613 | 0.317 | 0.005 | 0.251 | 0.006 |
| Histological subtype | 0.767 | 0.347 | 0.396 | 0.360 | 0.312 | 0.163 | 0.632 | 0.557 |
| Histological grade | 1.485 | 0.154 | 1.673 | 0.128 | 0.92 | 0.881 | 1.030 | 0.966 |
| FIGO stage (III/IV vs. I/II) | **4.637** | **4.02e-06** | **3.258** | **2.00e-03** | 1.339 | 0.660 | 1.887 | 0.393 |
| Debulking surgery outcome (≤1cm vs. >1cm RD) | **0.184** | **1.2 e-12** | **0.308** | **5.98e-06** | 1.581 | 0.328 | 1.178 | 0.757 |
| ***Immunofluorescence biomarker*** |  |  |  |  |  |  |  |  |
| Number of vessels* | **1.110** | **0.015** | 1.095 | 0.070 | 1.038 | 0.679 | 1.153 | 0.182 |
| Vessel density | **1.111** | **0.027** | 1.080 | 0.163 | 1.052 | 0.598 | 1.180 | 0.147 |
| Median vessel size | 1.001 | 0.325 | 1.015 | 0.752 | 0.003 | 0.999 | 1.003 | 0.236 |
| VEGFR-2 expression | **1.006** | **0.027** | 1.002 | 0.289 | 1.003 | 0.800 | 1.012 | 0.342 |
| c-MET/VEGFR-2 co-localisation | **1.011** | **0.003** | 1.004 | 0.186 | 1.018 | 0.159 | **1.040** | **0.001** |

**Supplementary Table 2. Prognostic and predictive biomarkers assessed as significant in the univariable analysis.** Key: *log2 transformed; HR, hazard ratio; RD, residual disease; PFS, progression-free survival; OS, overall survival. Multiple comparison was not adjusted for. Prognostic model, a model exploring if there is a significant association between a biomarker and survival independent of treatment arm; Predictive model, a model exploring if the association between a biomarker and survival are significantly different depending on treatment arms; HR and p-values in the predictive model comes from the interaction term.

| **SNP** | **Association with c-MET/VEGFR-2 co-localisation** | **Prognostic model** | | **Predictive model** | |
| --- | --- | --- | --- | --- | --- |
|  |  | **P-value for PFS** | **P-value for OS** | **P-value for PFS** | **P-value for OS** |
| ***VEGFA*** |  |  |  |  |  |
| rs2010963 | 0.729 | - | - | - | - |
| rs3025000 | 0.889 | - | - | - | - |
| rs3025030 | 0.314 | - | - | - | - |
| rs3025033 | **0.138** | 0.444 | 0.253 | 0.454 | 0.625 |
| rs3025035 | 0.851 | - | - | - | - |
| rs3025039 | 0.346 | - | - | - | - |
| rs6921438 | 0.281 | - | - | - | - |
| rs699946 | 0.391 | - | - | - | - |
| rs699947 | 0.326 | - | - | - | - |
| rs833058 | 0.217 | - | - | - | - |
| rs833060 | 0.594 | - | - | - | - |
| rs833061 | 0.283 | - | - | - | - |
| rs833068 | 0.858 | - | - | - | - |
| ***VEGFR-2*** |  |  |  |  |  |
| rs10020464 | 0.679 | - | - | - | - |
| rs11133360 | 0.273 | - | - | - | - |
| rs1411924 | 0.894 | - | - | - | - |
| rs1531289 | 0.472 | - | - | - | - |
| rs1531290 | 0.318 | - | - | - | - |
| rs17085262 | 0.268 | - | - | - | - |
| rs17085326 | 0.641 | - | - | - | - |
| rs1870377 | **0.151** | 0.616 | 0.091 | 0.552 | 0.994 |
| rs2034965 | 0.669 | - | - | - | - |
| rs2071559 | 0.990 | - | - | - | - |
| rs2125489 | 0.586 | - | - | - | - |
| rs2239702 | 0.360 | - | - | - | - |
| rs2305945 | **0.047** | 0.813 | 0.094 | 0.005 | 0.129 |
| rs2305948 | 0.839 | - | - | - | - |
| rs2305949 | 0.762 | - | - | - | - |
| rs34231037 | 0.829 | - | - | - | - |
| rs7667298 | 0.802 | - | - | - | - |
| rs7673274 | 0.474 | - | - | - | - |
| rs7691507 | 0.237 | - | - | - | - |
| rs12502008 | 0.664 | - | - | - | - |
| rs12505758 | 0.220 | - | - | - | - |
| rs12642307 | 0.707 | - | - | - | - |

**Supplementary Table 3. List of the 35 SNPs in *VEGF-A* and *VEGFR-2* tested and their association with c-MET/VEGFR-2 co-localisation.** Prognostic model, a model exploring if there is a significant association between a biomarker and survival independent of treatment arm; Predictive model, a model exploring if the association between a biomarker and survival are significantly different depending on treatment arms. P-values in the predictive model come from the interaction term.

| **SNP** | **Variant** | **ICON7 group** | |
| --- | --- | --- | --- |
|  |  | **C+P**  **(N=216)** | **C+P+B**  **(N=233)** |
| *VEGF-A* rs3025033 | A/A | 148 | 170 |
|  | A/G | 56 | 51 |
|  | G/G | 4 | 4 |
|  | *Total* | *208* | *225* |
| *VEGF-R2* rs1870377 | A/A | 118 | 125 |
|  | A/T | 81 | 96 |
|  | T/T | 16 | 11 |
|  | *Total* | *215* | *232* |
| *VEGFR-R2* rs2305945 | G/G | 83 | 93 |
|  | G/T | 108 | 104 |
|  | T/T | 23 | 34 |
|  | *Total* | *214* | *231* |

**Supplementary Table 4. Frequency of three key SNPs in VEGF-related genes.** Key: C, carboplatin; P, Paclitaxel; B, Bevacizumab. Data are presented as number of patients.

*Progression-free survival*, P=0.071

|  | **rs2305945 allele G** | **rs2305945 allele T** |
| --- | --- | --- |
| **c-MET/VEGFR-2 co-localisation increase** | 2.95 | 2.29 |
| **c-MET/VEGFR-2 co-localisation reduce** | 2.01 | 1 |

*Overall survival*, P=0.272

|  | **rs2305945 allele G** | **rs2305945 allele T** |
| --- | --- | --- |
| **c-MET/VEGFR-2 co-localisation increase** | 2.04 | 1.31 |
| **c-MET/VEGFR-2 co-localisation reduce** | 2.03 | 1 |

**Supplementary Table 5. Relationship between c-MET/VEGFR-2 co-localisation and *VEGFR-2* rs2305945 SNP.** c-MET/VEGFR-2 co-localisation, *VEGFR-2* rs2305945 and their interactions were included in a same multivariable model for PFS and OS.
